# Differential functional role of Orai1 variants in constitutive Ca^2+^ entry and calcification in luminal breast cancer cells

**DOI:** 10.1016/j.jbc.2024.107786

**Published:** 2024-09-18

**Authors:** Alejandro Berna-Erro, Jose Javier Lopez, Isaac Jardin, Jose Sanchez-Collado, Gines M. Salido, Juan A. Rosado

**Affiliations:** Department of Physiology (Cellular Physiology Research Group), Institute of Molecular Pathology Biomarkers (IMPB), University of Extremadura, Caceres, Spain

**Keywords:** Orai1α, Orai1β, constitutive Ca^2+^ entry, SPCA2, breast cancer, calcification

## Abstract

Resting cytosolic Ca^2+^ concentration is tightly regulated to fine-tune Ca^2+^-dependent cellular functions. Luminal breast cancer cells exhibit constitutive Ca^2+^ entry mediated by Orai1 and the secretory pathway Ca^2+^-ATPase, SPCA2, which result in mammary microcalcifications that constitute a prognostic marker of mammary lesions. Two Orai1 isoforms have been identified, the full-length Orai1α, consisting of 301 amino acids, and the short variant, Orai1β, lacking the 63 or 70 N-terminal amino acids comprising residues involved in channel inactivation and binding sites with Orai1 partners. We show that only the mammalian-specific Orai1α rescues SPCA2-dependent constitutive Ca^2+^ entry in Orai1-KO MCF7 cells, a widely used luminal breast cancer cell line. FRET analysis and immunoprecipitation revealed that Orai1α shows a greater ability to interact with SPCA2 than Orai1β. Deletion of the first 38 amino acids in Orai1α reduced the interaction with SPCA2 to a similar extent as Orai1β, thus suggesting that the N-terminal 38 amino acids play a relevant role in Orai1α-SPCA2 interaction. Finally, Orai1α, but not Orai1β, rescue the ability of Orai1-deficient cells to form *in vitro* microcalcifications. These findings provide compelling evidence for a functional role of Orai1α in constitutive Ca^2+^ entry in MCF7 cells, which might be a target to prevent the development of mammary microcalcifications in luminal breast cancer.

Store-operated calcium (Ca^2+^) entry (SOCE) is a major mechanism for Ca^2+^ influx in nonexcitable and, to a lesser extent, in excitable cells (1). SOCE generates Ca^2+^ signals that participate in many cellular functions such as migration, proliferation, division, and gene transcription (2, 3). The absence or the defective function of the molecular components of SOCE leads to serious, even fatal, clinical syndromes (2, 3). Remodeling of Ca^2+^ homeostasis in general, and SOCE in particular, have also been postulated as a prerequisite to support the altered physiology of cancer cells (4). SOCE is triggered by depletion of Ca^2+^ pools mainly located in the lumen of the endoplasmic reticulum (ER). Depleted Ca^2+^ stores are sensed by ER-resident transmembrane proteins STIM1 and STIM2, which undergo conformational changes that enable the interaction with and activation of plasma membrane (PM)-resident Orai channels (1). The Orai family comprises three isoforms: Orai1, Orai2, and Orai3. Orai1 channels generate highly selective Ca^2+^ currents (*I*_CRAC_, from Ca^2+^ release-activated Ca^2+^ current) and, with the participation of TRPC1, are involved in the activation of less Ca^2+^ selective store-operated currents (*I*_SOC_) (5, 6, 7). Two Orai1 variants have been identified so far, the most studied Orai1α of 301 amino acids, and Orai1β, which is generated by an alternative initiation site located in the coding sequence of the mRNA, giving rise to a shorter version lacking the N-terminal 63 amino acids (8, 9). Both variants exhibit different biophysical and functional properties. Among them, Orai1β is less sensitive to Ca^2+^-dependent inactivation (8, 9) and, while Orai1α is required for the PM location and activation TRPC1 channels, the role of Orai1β in this process is cell specific (10). Furthermore, Orai1β lacks the protein kinase C (PKC; Ser27, and Ser30 in Orai1α) and protein kinase A (PKA; Ser34 in Orai1α) phosphorylation sites, which makes Orai1β less sensitive to phosphorylation-dependent inactivation (1). The absence of an interaction motif for Ca^2+^-activated adenylyl cyclase 8 prevents Orai1β from participating in cAMP signaling pathways, which is relevant for cancer cell biology (11, 12). The lack of the AKAP79 binding motif prevents Orai1β from stimulating the nuclear factor-κB-dependent transcriptional activity (13). Finally, only Orai1α exhibit binding regions for phosphatidylinositol 4,5-bisphosphate and caveolin in the N-terminal region, which might be relevant for the subcellular distribution (1, 14). Despite these differences both variants are equally efficient mediating the *I*_CRAC_ current (8), but only Orai1α is involved in the store-independent arachidonic acid-triggered Ca^2+^ currents (*I*_ARC_) (8, 15).

Orai1 also associates with the small conductance Ca^2+^-activated K^+^ channel 3 (SK3) or the Golgi-resident secretory pathway Ca^2+^-ATPase 2 (SPCA2) to generate constitutive Ca^2+^ currents, which are important for proliferation, migration, and tumorigenesis of cancer cells (16, 17). Feng *et al.* found increased cytosolic free-Ca^2+^ concentration ([Ca^2+^]_C_) associated with overexpressed SPCA2 in estrogen receptor positive (ER+) breast cancer cells (17). Silencing of SPCA2 in MCF7 ER+ breast cancer cells decreases constitutive Ca^2+^ entry and reduces [Ca^2+^]_C_, indicating a link between both events. They found that such an increased [Ca^2+^]_C_ is mediated by Orai1 currents, which were constitutively activated by SPCA2. Thus, the N and C termini of both proteins interact with each other in a sequential manner that leads to constitutive Orai1 activation and Ca^2+^ influx. The SPCA2/Orai1 association does not depend on STIM1 and does not alter either ER Ca^2+^ stores or SOCE, suggesting a separate pathway from SOCE mediated by different Orai1 subpopulations. SPCA2 knockdown in these cells impairs proliferation, reduces the *in vitro* formation of extracellular microcalcifications, and inhibits tumor generation in nude mice injected with MCF7 SPCA2-knowdown cells, indicating a critical role of this SPCA2/Orai1 interaction in cancer cell biology (17, 18). This interaction was later reported to be crucial for Ca^2+^ transportation by epithelial mammary cells during lactation, since SPCA2 couples the Orai1-mediated Ca^2+^ influx with the Golgi secretory pathway (19, 20). Here, we have investigated the involvement of Orai1α and Orai1β in constitutive Ca^2+^ entry in ER+ breast cancer cells. Our results indicate that Orai1α exhibits a greater efficiency than Orai1β interacting with SPCA2 and mediating this pathway, which is likely mediated by the first 38 amino acids. As a result, Orai1α is the only Orai1 variant responsible for *in vitro* calcification mediated by SPCA2 in MCF-7 cells, which suggest that Orai1α might be considered as a specific therapeutic target in the treatment of ER+ breast cancer.

## Results

### Rescue of constitutive Ca^2+^ entry in Orai1-KO MCF7 cells

**Figure 1:**
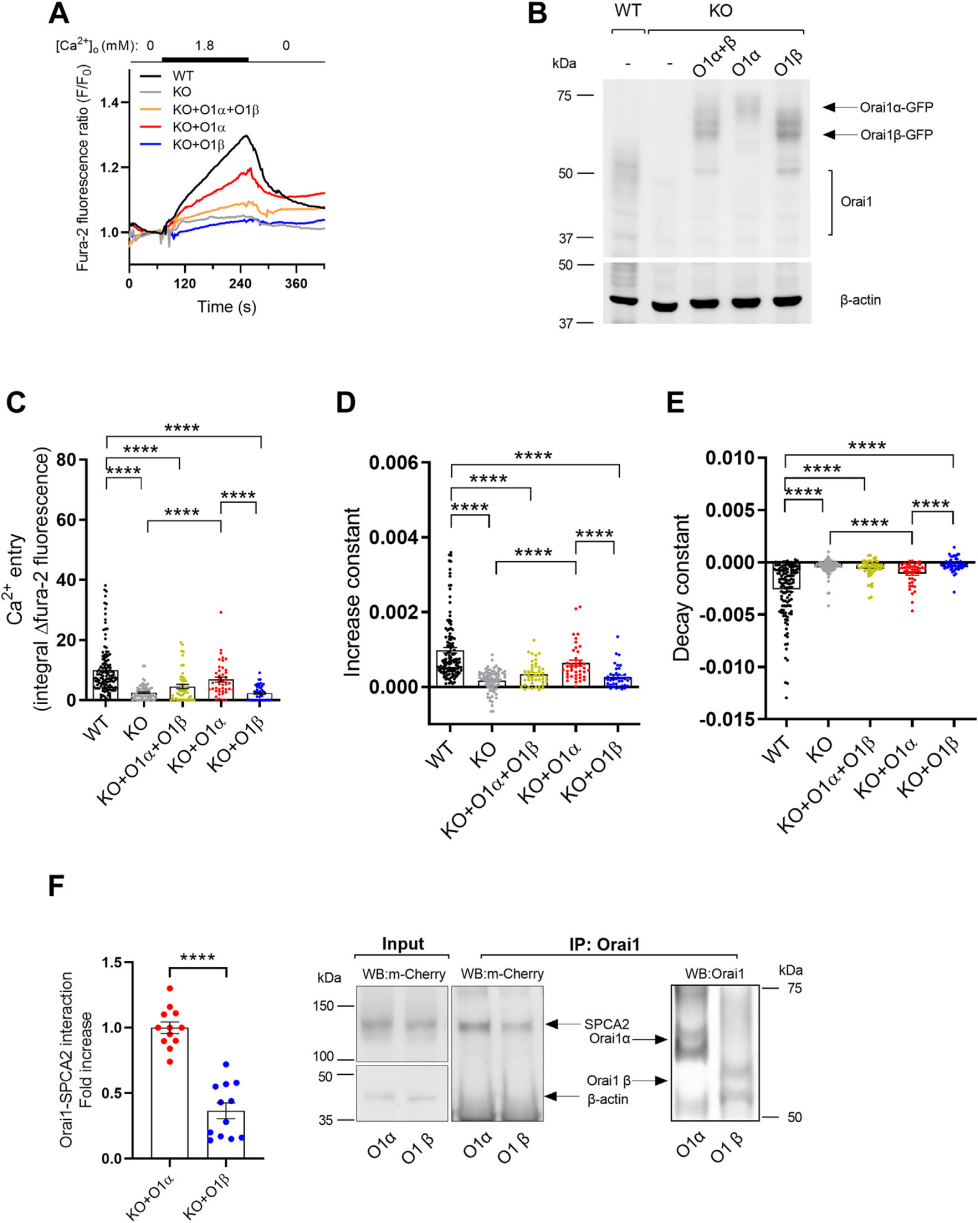
**Role of Orai1α and Orai1β in constitutive Ca**^**2+**^**entry in MCF7 cells.***A* and *B*, WT MCF7 cells (WT), Orai1-KO MCF7 cells (KO) and KO cells transfected with TK promoter plasmids either for Orai1α-eGFP (O1α), Orai1β-eGFP (O1β), or both (O1α+O1β), were either loaded with fura-2 *A* or lysed *B*. *A*, Fura-2-loaded cells were perfused with a Ca^2+^-free HBS (100 μM EGTA added), followed by perfusion with HBS containing 1.8 mM Ca^2+^ and then further perfused with a Ca^2+^-free HBS (1 mM EGTA added). Graphs represent mean values. *B*, cell lysates were subjected to 10% SDS-PAGE and Western blotting with the anti-Orai1 antibody, as described in Experimental procedures. Membranes were reprobed with anti-β-actin antibody for protein loading control. Molecular masses indicated on the *left* were determined using molecular-mass markers run in the same gel. *C*, quantification of Ca^2+^ entry was determined as described in Experimental procedures. Bar graphs are represented as mean ± SEM and expressed as the integral of the rise in fura-2 fluorescence ratio after the addition of extracellular Ca^2+^ and taking a sample every second. From left to right, n = 127, 77, 50, 43, and 47; n values correspond to individual cells. Data were statistically analyzed using Kruskal–Wallis test with multiple comparisons (Dunn's test). ∗∗∗∗*p* < 0.0001. *D* and *E*, quantification of the increase (*D*), and decay (b) constants (*E*), for all the conditions presented in (*A*) estimated as described in Experimental procedures. Data are presented as mean ± SEM. Data were statistically analyzed using Kruskal–Wallis test with multiple comparisons (Dunn's test). ∗∗∗∗*p* < 0.0001. *F*, KO cells were transfected with TK promoter driven plasmids for O1α or O1β and mCherry-SPCA2. Forty-eight hours later, cells were lysed and whole cell lysates were either immunoprecipitated with anti-Orai1 antibody or analyzed by Western blotting with anti-mCherry antibody (input). Immunoprecipitates were subjected to 10% SDS-PAGE and Western blotting with the anti-mCherry antibody, as described in Experimental procedures. Membranes were reprobed with the antibody used for immunoprecipitation for protein loading control. Data are presented as mean ± SEM. Twelve separate experiments were statistically analyzed using unpaired *t* test. ∗∗∗∗*p* < 0.0001. Molecular masses were determined using molecular-mass markers run in the same gel. HBS, Hepes-buffered saline; SPCA2, secretory pathway Ca^2+^-ATPase 2; TK, thymidine kinase.

Previous studies reported the role of Orai1 in constitutive Ca^2+^ entry in breast cancer cells (17). However, the identification of two Orai1 variants, Orai1α and Orai1β, makes it necessary to revisit this event to analyze the relevance of both Orai1 forms in this process. Therefore, we have evaluated the participation of Orai1α and Orai1β in this process. The alignment of the amino acid sequences of both Orai1 variants is depicted in Fig. S1. As shown in Figure 1, *A*, *C*–*E*, fura-2-loaded MCF-7 cells were initially perfused with a Ca^2+^-free medium (100 μM EGTA added) and subsequent perfusion with a medium containing 1.8 mM Ca^2+^ raised the fura-2 fluorescence ratio in resting cells to 1.3-fold, which is indicative of constitutive Ca^2+^ entry, the fura-2 fluorescence ratio decreased again upon perfusion with a Ca^2+^-free medium (1 mM EGTA added), which further confirms that the rise in fura-2 fluorescence is mediated by Ca^2+^ influx. The evaluation of the area under the curve gave an estimation of Ca^2+^ entry (Fig. 1*C*). Interestingly, constitutive Ca^2+^ entry was almost completely abolished in Orai1-KO MCF7 cells, which further supports the role of Orai1 in this mechanism (Fig. 1, *A* and *C*). In order to explore the relevance of the Orai1 forms in constitutive Ca^2+^ entry Orai1-KO MCF7 cells were transfected with thymidine kinase-driven Orai1α and/or β. Expression of the different Orai1 forms was confirmed by in Figure 1*B*. Western blotting of WT cell lysates with anti-Orai1 antibody resulted in several diffuse bands around and over 37 kDa which has been attributed to Orai1 N-glycosylation (as these multiple bands disappeared following treatment of cell lysates with N-glycosidase F (PNGase F), which hydrolyzes glycosylamine linkage of asparagine-linked oligosaccharides) (9, 13) and, perhaps, also to Orai1 Ser-phosphorylation (as Orai1 has been reported to be phosphorylated as Ser residues 27, 30, and 34) (21). Expression of Orai1α-GFP or Orai1β-GFP led to diffuse bands with the expected size of GFP fused Orai1 variants (Figs. 1*B* and S3, uncropped gel images). Expression of Orai1α almost completely restored constitutive Ca^2+^ influx giving a fura-2 fluorescence elevation that was not significantly different to WT cells (Fig. 1, *A* and *C*); however, Orai1β failed to restore constitutive Ca^2+^ influx providing a signal that was similar to that observed in Orai1-KO MCF7 cells (Fig. 1, *A* and *C*). Of note, cotransfection of Orai1α with Orai1β significantly attenuated the extent of constitutive Ca^2+^ entry as compared to transfection of Orai1α alone, despite, we used the same amount of total Orai1 plasmid in both conditions, suggesting that Orai1β might modulate constitutive Ca^2+^ entry mediated by Orai1α and SPCA2 in these cells. Analysis of the increase and decay constants (Fig. 1, *D* and *E*; n = 43–127) revealed proportional values to the area under the curve, indicative of unchanged dynamics of Ca^2+^ entry in all conditions (the increase constants were 9.82 × 10^4^ ± 7.57 × 10^5^, 1.66 × 10^4^ ± 3.29 × 10^5^, 3.38 × 10^4^ ± 4.59 × 10^5^, 6.35 × 10^4^ ± 7.65 × 10^5^, and 2.61 × 10^4^ ± 4.62 × 10^5^ for WT cells, Orai1-KO cells, and Orai1-KO cells expressing either Orai1α and Orai1β, Orai1α or Orai1β, respectively; and the decay constants were −2.59 × 10^3^ ± 2.35 × 10^4^, −4.60 × 10^4^ ± 8.31 × 10^5^, −5.94 × 10^4^ ± 1.23 × 10^4^, −1.07 × 10^3^ ± 1.63 × 10^4^, and −2.28 × 10^4^ ± 9.53 × 10^5^ for WT cells, Orai1-KO cells and Orai1-KO cells expressing either Orai1α and Orai1β, Orai1α or Orai1β, respectively).

### Interaction of SPCA2 with Orai1 variants

Previous studies reported that SPCA2 directly interacts with Orai1α, triggering the activation of constitutive Ca^2+^ entry in MCF7 breast cancer cells (17). We hypothesized then that an abrogated SPCA2-Orai1β interaction must be responsible of its lack of function. Therefore, we have analyzed the Orai1/SPCA2 interaction in Orai1-KO MCF7 cells expressing mCherry-SPCA2 together with Orai1α-enhanced GFP (eGFP) or Orai1β-eGFP by immunoprecipitation and Western blotting. In Orai1α expressing cells, immunoprecipitation with anti-Orai1 antibody followed by Western blotting with an anti-mCherry antibody revealed the presence of a 125 kDa immunoreactive band compatible with the estimated size of the mCherry-SPCA2 fusion protein (Fig. 1*F*), suggesting that Orai1α interacts with SPCA2. Consistent with the Ca^2+^ experiments, in cells expressing Orai1β, detection of SPCA in Orai1 immunoprecipitates was reduced by 63% as compared to Orai1α (Fig. 1*F*; n = 12), indicating defective interaction between Orai1β and SPCA that explains the abrogated constitutive Ca^2+^ entry observed in Orai1-KO MCF7 cells expressing Orai1β (Fig. 1, *A* and *C*).

**Figure 2:**
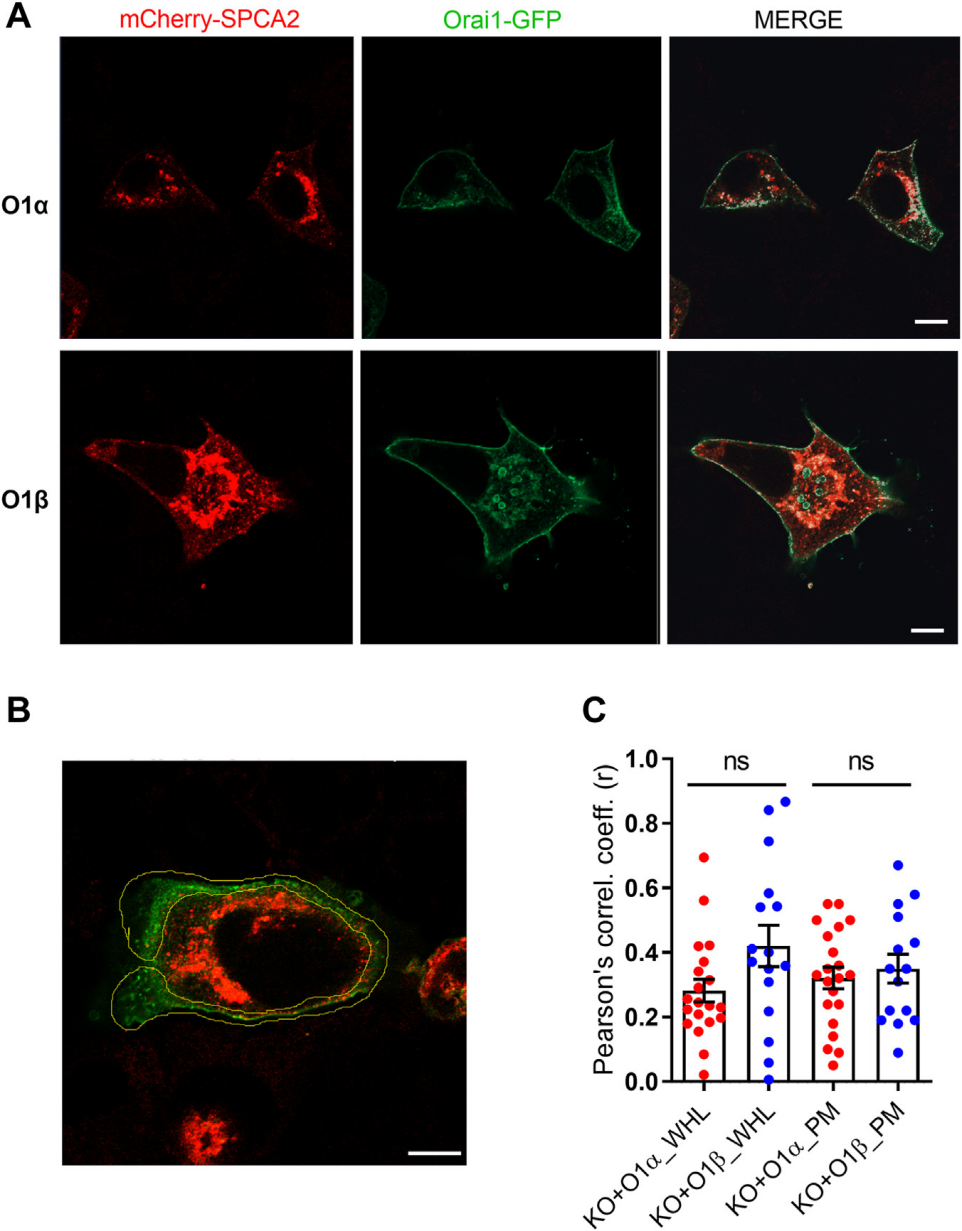
**Colocalization between the Orai1 variants and SPCA2.***A*, confocal images of MCF7 cells transfected with CMV promoter mCherry-SPCA2 and either Orai1α-eGFP (O1α) or Orai1β-eGFP (O1β), as indicated. Images of Orai1-eGFP (*green*), mCherry-SPCA2 (*red*), or merged images were taken in nonstimulated cells. Overlapped pixels are shown in *white* in the merge panels. The scale bar represents 10 μm. *B*, representative image of the membrane-specific ROI used to study colocalization in the PM. The scale bar represents 10 μm. *C*, Pearson correlation coefficients obtained with the plot profile analysis of O1α or O1β channels with mCherry-SPCA2 in the whole cell (WHL) or the PM (PM). Mean ± S.E.M. 16 to 20 separated experiments were statistically analyzed using the Mann-Whitney U-test. CMV, cytomegalovirus; PM, plasma membrane; ROI, region of interest; SPCA2, secretory pathway Ca^2+^-ATPase 2.

**Figure 3:**
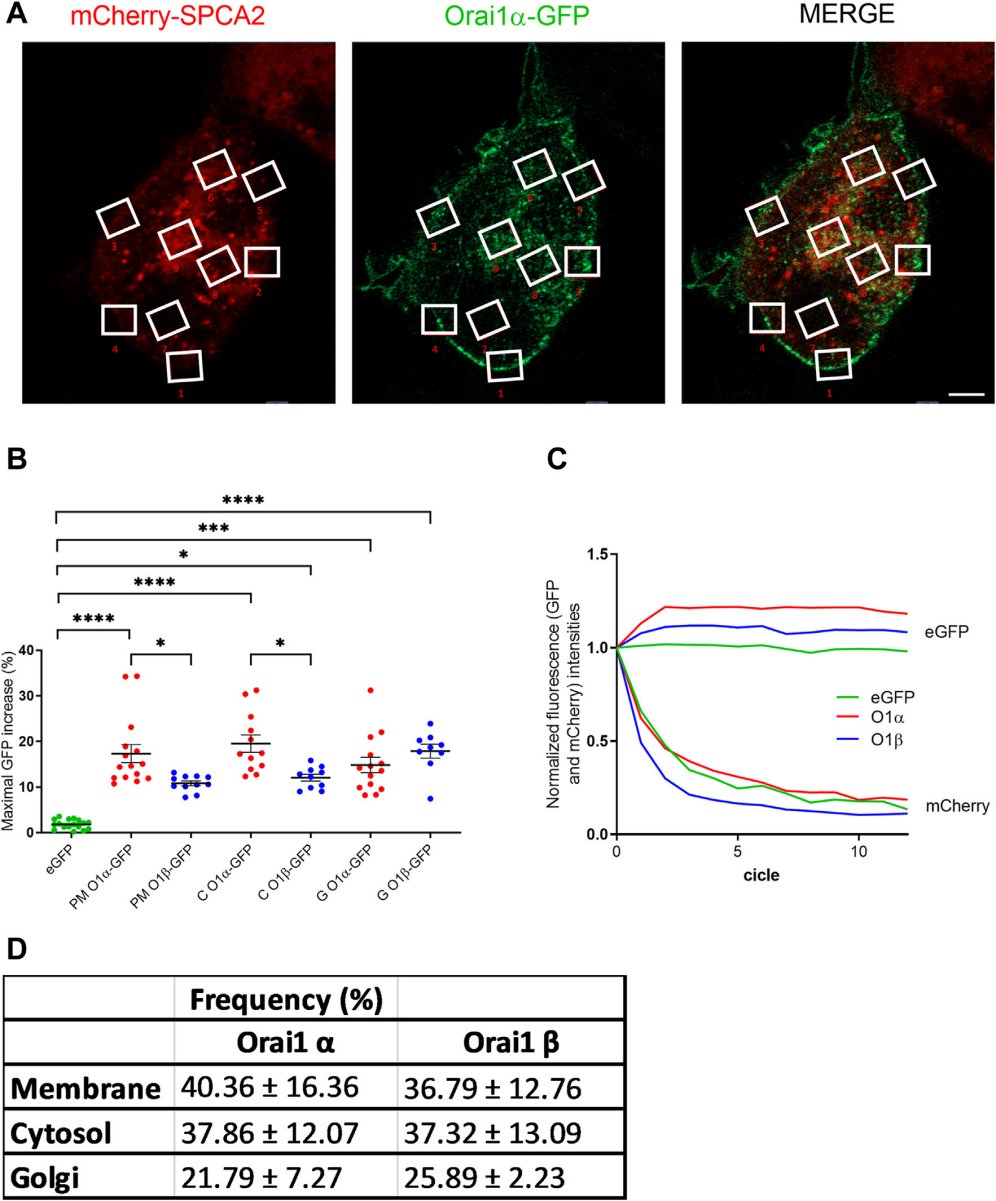
**Interaction of Orai1 variants with SPCA2.***A*, MCF7 cells were cotransfected with CMV driven mCherry-SPCA2 and either free eGFP, Orai1α-eGFP (O1α) or Orai1β-eGFP (O1β). Images show mCherry-SPCA2 (in *red*), Orai1α-eGFP (in *green*) and the merged image. ROIs indicate the places where FRET was analyzed. The scale bar represents 10 μm. *B*, scatter plots represent the maximal high FRET efficiencies corresponding to free eGFP, O1α, or O1β and mCherry-SPCA2 interaction in the plasma membrane (PM), cytosol (C) and the Golgi (G). Mean ± S.E.M. From *left* to *right*, n = 17, 15, 11, 12, 10, 14, 9 FRET events in 28 and 26 separate cells for free eGFP, O1α and O1β, respectively, were statistically analyzed using the Kruskal–Wallis test. ∗*p* < 0.05, ∗∗∗*p* < 0.001 and ∗∗∗∗*p* < 0.0001. *C*, representative graphs of normalized fluorescence intensities from cytosolic free eGFP (*green*), O1α (red) and O1β (*blue*) donors and their respective SPCA2-mCherry acceptors registered during 13 cycles of an acceptor photobleaching FRET experiment. *D*, the frequency of FRET events at the PM, cytosol, and Golgi region was determined as described in Experimental procedures and shown in the table as Mean ± S.E.M. CMV, cytomegalovirus; PM, plasma membrane; ROI, region of interest; SPCA2, secretory pathway Ca^2+^-ATPase 2.

To confirm this possibility, we further analyzed the Orai1 variant/SPCA2 interaction by colocalization in resting MCF7 KO cells overexpressing mCherry-SPCA2 and either Orai1α-eGFP or Orai1β-eGFP. Confocal images revealed a basal colocalization of SPCA2 and Orai1α in resting conditions as described elsewhere (Fig. 2) (17). Even though both proteins were widely expressed, colocalization was restricted to Golgi and PM, suggesting that only a tiny subset of Orai1 and SPCA2 interacted with one another (Fig. 2*A*). Pearson score analysis did not reveal any significant difference between the Orai1 forms in colocalization with SPCA2 in the whole cell (Fig. 2*C*; n = 16–21). Next, we studied protein colocalization in the membrane, setting a region of interest (ROI) around the PM (Fig. 2, *B* and *C*). No significant difference was found among the colocalization of both Orai1 variants and SPCA2 in the PM, indicating that Orai1α and Orai1β equally colocalize with SPCA2. However, the minimal spherical particle radii of proteins within 20 to 100 kDa mass, such as GFP and mCherry for Orai1 and SPCA2, respectively, ranges within 1.78 to 3.05 nm (22), and our confocal setup has a limited optical resolution of 160 nm. This means that all overlapping and nonoverlapping fluorescence signals separated by less than that are considered to be overlapped since the setup cannot discriminate between them. FRET allows the detection of intermolecular distances lower than 10 nm, whose efficiency varies with the sixth power of the distance between the donor and the acceptor (23). Therefore, we examined FRET in nine ROIs located in the cytosol, the Golgi, and PM to detect tightly interacting Orai1 and SPCA2 proteins (Fig. 3*A*). FRET analysis revealed that mCherry-SPCA2 and both Orai1-eGFP interact with one another close enough to allow energy transfer as compared to free eGFP (Fig. 3*B*), indicating a direct interaction between both proteins as previously suggested elsewhere (17, 19). Furthermore, our results indicate that PM and cytosolic Orai1β-eGFP was less efficient at generating FRET than Orai1α-eGFP (Fig. 3*B*; from left to right, n = 15, 11, 12, 10), indicating a significantly higher molecular distance with SPCA2 than that of Orai1α. However, there was no significant differences between mCherry-SPCA2 and both Orai1-eGFP located in the Golgi (Fig. 3*B*; n = 14, 9), probably indicating a poor interaction at this organelle. Once more, we found FRET in only one or a few ROIs in each cell, indicating that their interaction is restricted to very localized regions inside the cell. In fact, we found variations in the frequency of FRET events observed at different subcellular regions (Fig. 3*D*). Orai1 variants showed equal frequencies of FRET events in the PM, while Orai1α showed slightly increased FRET frequencies in the cytosol, but less FRET events in the Golgi as compared to Orai1β. In conclusion, Orai1β seems to not participate in constitutive Ca^2+^ entry due to a weak but conserved interaction with SPCA2, characterized by an increased distance between both proteins that presumably impedes Orai1β activation.

### Role of the N-terminal 38 amino acids of Orai1α in constitutive Ca^2+^ entry

**Figure 4:**
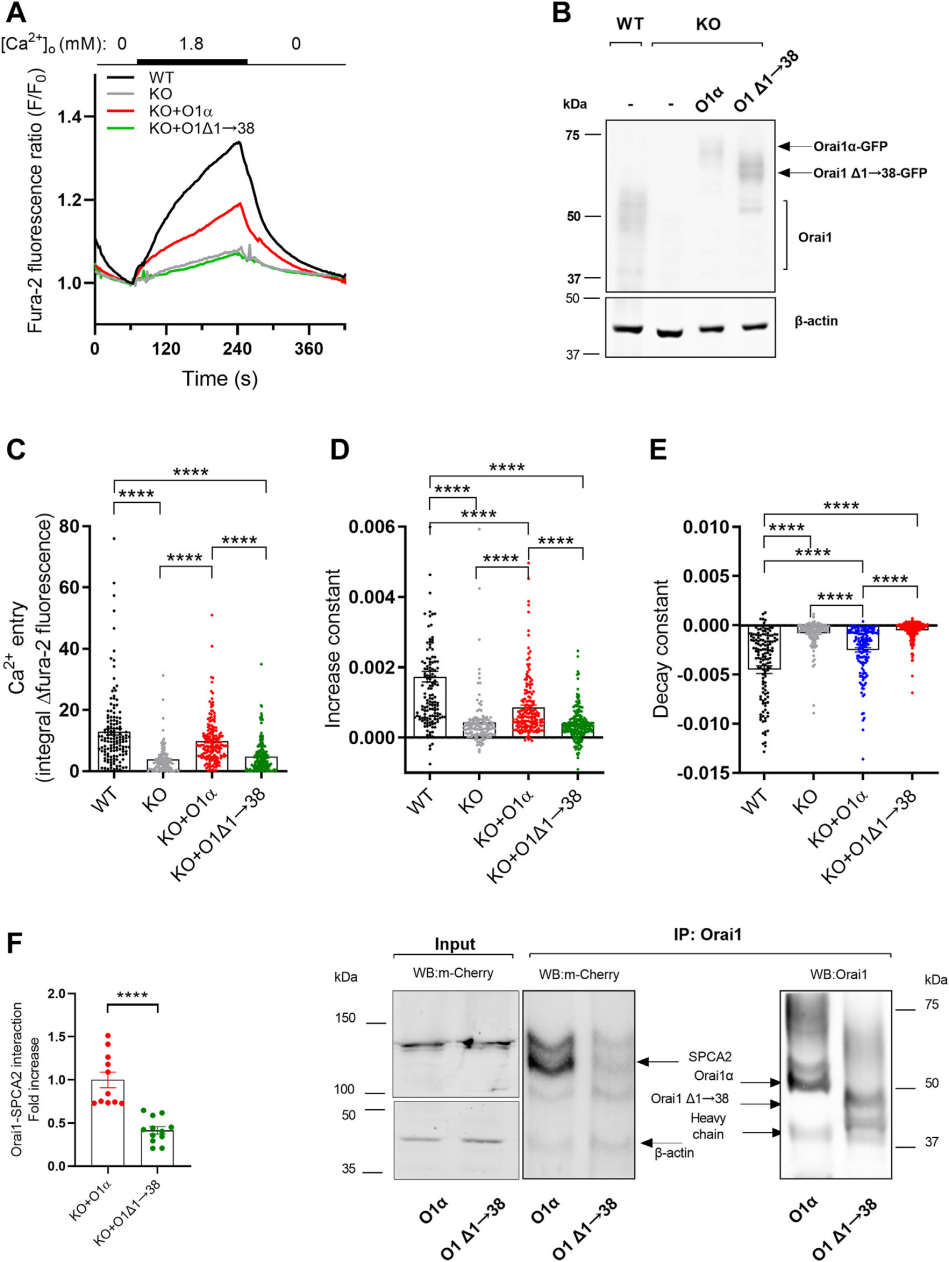
**Role of Orai1α and its N-terminal 38 amino acid region in constitutive Ca**^**2+**^**entry in MCF7 cells.***A* and *B*, WT MCF7 cells (WT), Orai1-KO MCF7 cells (KO) and KO cells transfected with CMV driven Orai1α-eGFP (O1α) or Orai1αΔ1-38 (O1Δ1→38) were either loaded with fura-2 *A* or lysed *B*. *A*, Fura-2-loaded cells were perfused with a Ca^2+^-free HBS (100 μM EGTA added), followed by perfusion with HBS containing 1.8 mM Ca^2+^ and then further perfused with a Ca^2+^-free HBS (1 mM EGTA added). *B*, cell lysates were subjected to 10% SDS-PAGE and Western blotting with the anti-Orai1 antibody, as described in Experimental procedures. Membranes were reprobed with anti-β-actin antibody for protein loading control. Molecular masses indicated on the left were determined using molecular-mass markers run in the same gel. *C*, quantification of Ca^2+^ entry was determined as described in Experimental procedures. Bar graphs are represented as mean ± SEM and expressed as the integral of the rise in fura-2 fluorescence ratio after the addition of extracellular Ca^2+^ and taking a sample every second. From *left* to *right*, n = 142, 123, 172, and 204; n values correspond to individual cells. Data were statistically analyzed using Kruskal–Wallis test with multiple comparisons (Dunn's test). ∗∗∗∗*p* < 0.0001. *D* and *E*, quantification of the increase *D*, and decay constants *E*, for all the conditions presented in A estimated as described in Experimental procedures. Data are presented as mean ± SEM. Data were statistically analyzed using Kruskal–Wallis test with multiple comparisons (Dunn's test). ∗∗∗∗*p* < 0.0001. *F*, KO cells were transfected with CMV driven plasmids for O1α or O1Δ1→38 and mCherry-SPCA2. Forty-eight hours later, cells were lysed and whole cell lysates were either immunoprecipitated with anti-Orai1 antibody or analyzed by Western blotting with anti-mCherry antibody (input). Immunoprecipitates were subjected to 10% SDS-PAGE and Western blotting with the anti-mCherry antibody, as described in Experimental procedures. Membranes were reprobed with the antibody used for immunoprecipitation for protein loading control. Molecular masses were determined using molecular-mass markers run in the same gel. Data are presented as mean ± SEM. Eleven separate experiments were statistically analyzed using unpaired *t* test. ∗∗∗∗*p* < 0.0001. CMV, cytomegalovirus; HBS, Hepes-buffered saline; O1Δ1→38, N-terminal deletion mutant Orai1α ΔN1→38; SPCA2, secretory pathway Ca^2+^-ATPase 2.

**Figure 5:**
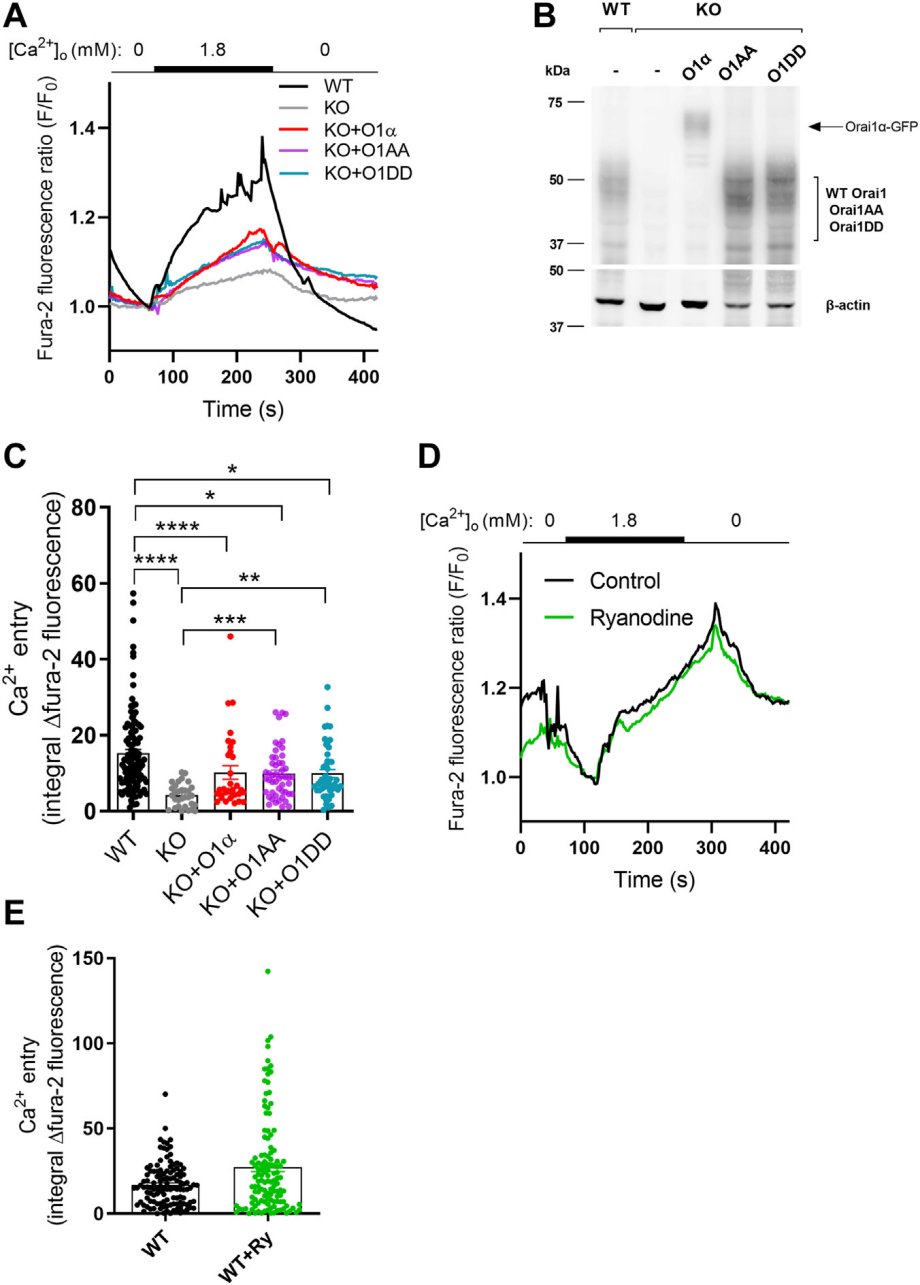
**Role of Orai1α phosphorylation at Ser-27 and Ser-30 and ryanodine receptors in constitutive Ca**^**2+**^**entry in MCF7 cells.***A–C*, WT MCF7 cells (WT), Orai1-KO MCF7 cells (KO), and KO cells transfected with CMV promoter plasmids either for Orai1α (O1α), Orai1α-S27/30A (O1AA) or Orai1α-S27/30D (O1DD), were either loaded with fura-2 *A* or lysed *B*. *A*, Fura-2-loaded cells were perfused with a Ca^2+^-free HBS (100 μM EGTA added), followed by perfusion with HBS containing 1.8 mM Ca^2+^ and then further perfused with a Ca^2+^-free HBS (1 mM EGTA added). Graphs represent mean values. *B*, cell lysates were subjected to 10% SDS-PAGE and Western blotting with the anti-Orai1 antibody, as described in Experimental procedures. Membranes were reprobed with anti-β-actin antibody for protein loading control. Molecular masses indicated on the *left* were determined using molecular-mass markers run in the same gel. Blots are representative of four separate experiments. *C*, quantification of Ca^2+^ entry was determined as described in Experimental procedures. Bar graphs are represented as mean ± SEM and expressed as the integral of the rise in fura-2 fluorescence ratio after the addition of extracellular Ca^2+^ and taking a sample every second. From *left* to *right*, n = 116, 33, 37, 50, and 49; n values correspond to individual cells. Data were statistically analyzed using Kruskal–Wallis test with multiple comparisons (Dunn's test). ∗*p* < 0.05, ∗∗*p* < 0.01, ∗∗∗*p* < 0.001 and ∗∗∗∗*p* < 0.0001. *D–F*, Fura-2-loaded WT MCF7 cells were treated with 100 μM ryanodine for 30 min and, either were perfused with a Ca^2+^-free HBS (100 μM EGTA added), followed by perfusion with HBS containing 1.8 mM Ca^2+^ and then further perfused with a Ca^2+^-free HBS (1 mM EGTA added) *D* or lysed *E*. *D*, graphs represent mean values. *E*, quantification of Ca^2+^ entry was determined as described in Experimental procedures. Bar graphs are represented as mean ± SEM and expressed as the integral of the rise in fura-2 fluorescence ratio after the addition of extracellular Ca^2+^ and taking a sample every second. From *left* to *right*, n = 280 and 154; n values correspond to individual cells. Data were statistically analyzed using Mann-Whitney U-test. CMV, cytomegalovirus; HBS, Hepes-buffered saline.

A previous study reported that both the N and C termini of Orai1 interact with SPCA2 (17). To better characterize the interaction site, we evaluated the function of an Orai1α truncated mutant lacking the first 38 amino acids at the N terminus of Orai1α (O1Δ1→38) as compared to Orai1α (Fig. S1). The expression of O1Δ1→38 or Orai1α was confirmed by Western blotting (Fig. 4*B*). Imaging analysis of Ca^2+^ homeostasis revealed that the O1Δ1→38 mutant failed to restore constitutive Ca^2+^ entry of Orai1-KO MCF7 cells as compared to Orai1α (Fig. 4, *A* and *C*; n = 127–204), similar to that observed in cells expressing Orai1β (Fig. 1, *A* and *C*). Analysis of the increase and decay constants revealed values that qualitatively were consistent with the area under the curve, indicative of unchanged dynamics of Ca^2+^ entry in all conditions (Fig. 4, *D* and *E*; the increase constants were 1.73 × 10^3^ ± 1.501 × 10^4^, 4.34 × 10^4^ ± 6.87 × 10^5^, 8.6 × 10^4^ ± 6.38 × 10^5^, and 3.69 × 10^4^ ± 3.19 × 10^5^ for WT cells, Orai1-KO cells and Orai1-KO cells expressing either Orai1α or Orai1Δ1→38, respectively; and the decay constants were −4.49 × 10^3^ ± 3.94 × 10^4^, −8.09 × 10^4^ ± 1.23 × 10^4^, −2.48 × 10^3^ ± 2.13 × 10^4^, and −5.13 × 10^4^ ± 6.61 × 10^5^ for WT cells, Orai1-KO cells, and Orai1-KO cells expressing either Orai1α or Orai1Δ1→38, respectively; n = 127–204). Immunoprecipitation with anti-Orai1 antibody followed by Western blotting with an anti-mCherry antibody revealed a decrease in SPCA2 detection by 58% as compared to Orai1α (Figs. 4*F* and S3 for uncropped gel images; n = 11). Thus, our results demonstrate that the N terminal 38 amino acids of Orai1α are required for the interaction with SPCA2. This region contains three phosphorylation sites: Ser27 and Ser30 are susceptible to be phosphorylated by PKC, and Ser34 is phosphorylated by PKA, which play a relevant role in Orai1 inactivation (1, 11, 21, 24). Therefore, we studied the role of PKC phosphorylation on constitutive Ca^2+^ entry in Orai1-KO MCF7 cells transfected with the nonphosphorylatable mutant of Orai1α on the S27 and S30 residues (Flag-Orai1-S27A/S30A (O1AA)), and the phosphomimetic Orai1α mutant Flag-Orai1-S27D/S30D (O1DD) (Fig. S1). Expression of Orai1α or the Orai1α mutants was confirmed by Western blotting (Figs. 5*B* and S3 for uncropped gel images). Imaging analysis of constitutive Ca^2+^ entry revealed a similar response in Orai1-KO cells expressing Orai1α or the Orai1α mutants O1AA and O1DD (Fig. 5, *A* and *C*; the integral of the rise in fura-2 fluorescence was 15.23 ± 1.01, 4.21 ± 0.54, 10.18 ± 1.75, 9.93 ± 0.92, and 9.93 ± 1.02 in WT cells, Orai1-KO cells or Orai1-KO cells expressing Orai1α or the Orai1α mutants O1AA and O1DD, respectively; n = 33–116). We further studied the role of PKA phosphorylation on constitutive Ca^2+^ entry in Orai1-KO MCF7 cells transfected with a nonphosphorylatable mutant of Orai1α on the S34 residue (Orai1-S34A-GFP (O1-34)). Imaging analysis of constitutive Ca^2+^ entry revealed a similar response again in Orai1-KO cells expressing Orai1α or the Orai1α mutant O1-34 (Fig. S2, *A* and *B*; n = 41, 36, 26, and 24). These findings indicate that phosphorylation at serines 27, 30, and 34 is not necessary for the activation of constitutive Ca^2+^ entry.

Saldaña *et al.* reported the expression of functional ryanodine receptors (RyRs) in the Golgi of MCF7 cells, which seems to contribute to the regulation of basal [Ca^2+^]_C_ in those cells (25). ER-resident RyR are involved in Ca^2+^-induced Ca^2+^ release from the ER compartment to the cytosol (26). Therefore, we wondered whether Golgi-resident RyR might have a similar role in Ca^2+^ homeostasis in MCF7 cells by playing a role in constitutive Ca^2+^ entry. However, Ca^2+^ imaging experiments revealed a similar constitutive Ca^2+^ entry in MCF7 WT cells treated with a high concentration of ryanodine (100 μM) or vehicle (Fig. 5, *D* and *E* n = 154–280), which strongly indicates that RyRs are not required for constitutive Ca^2+^ entry in MCF7 cells.

### Function of Orai1 variants in *in vitro* microcalcifications

**Figure 6:**
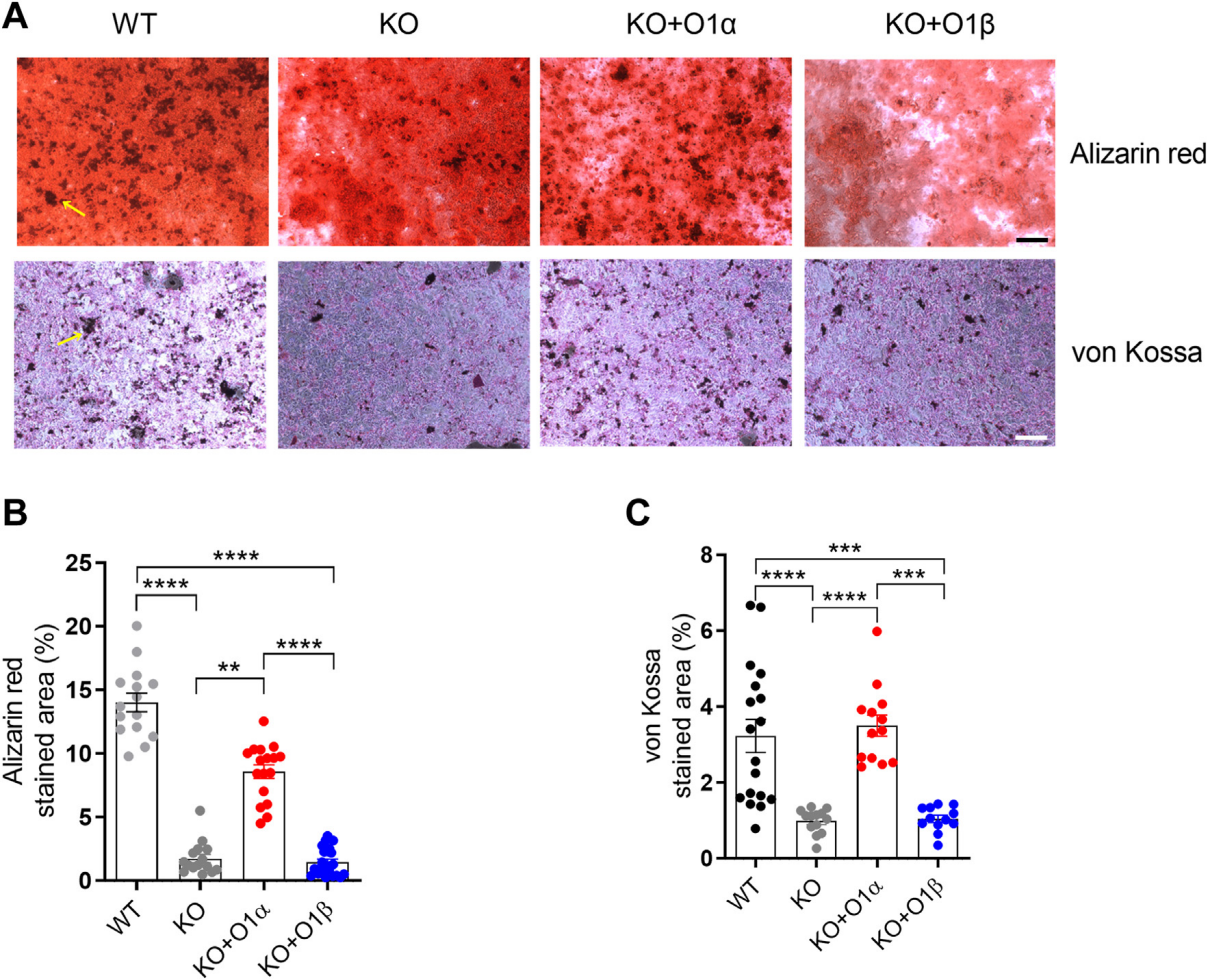
**Role of Orai1 variants in the Golgi/secretory pathway and in calcification in MCF7 cells.***A*, WT MCF7 cells (WT), Orai1-KO MCF7 cells (KO), and KO cells transfected with thymidine kinase promoter plasmids either for Orai1α-eGFP (O1α) or for Orai1β-eGFP (O1β), were grown to confluency and DMEM medium was supplemented with osteogenic media for 7 days. Cells were later fixed and stained with alizarin *red* (*upper panels*) or Von Kossa (*lower panels*) to highlight Ca^2+^ phosphate precipitates as specified in material and methods. *B* and *C*, microcalcifications stained in darker *red* (*yellow arrows*) for alizarin *red* and *black* for Von Kossa was quantified and plotted as bar graphs representing the mean ± SEM percentage of covered surface area. *Gray shapes* corresponding to apoptotic cell areas (*yellow asterisks*) were not included in the quantification. From *left* to *right*, *B*, n = 15, 15, 17, and 122; *C*, n = 18, 13, 13, and 12; n values correspond to total number of fields from four independent experiments. Data were statistically analyzed using Kruskal–Wallis test with multiple comparisons (Dunn's test). ∗∗*p* < 0.01, ∗∗∗*p* < 0.001 and ∗∗∗∗*p* < 0.0001. The scale bar represents150 μm. DMEM, Dulbecco's modified Eagle's medium.

Smaardijk *et al.* reported that SPCA2 couples Orai1-mediated constitutive Ca^2+^ entry with the Golgi/secretory pathway in human embryonic kidney HEK293 and adenocarcinoma HelaT1 cells, so that SPCA2 transfers the internalized cytosolic Ca^2+^ to the Golgi, which, in turn, is secreted to the extracellular compartment. This mechanism might have an important role in SPCA2-expressing secretory cells, such as epithelial mammary cells during lactation (20). Calcifications of breast carcinomas are frequently crystals of Ca^2+^ phosphates secreted by tumoral cells, deposited as hydroxyapatite in the extracellular matrix, and associated with a poorer prognosis in breast cancer patients (27). The radiologic study of their morphology is used as a marker for diagnosis. It has been proposed that the SPCA2-dependent secretory pathway might be important for calcifications of breast carcinomas, since the *in vitro* silencing of SPCA2 in MCF7 cells decreases the number of stromal microcalcifications (18). Therefore, we have further evaluated whether the Orai1 variants participate in the mechanism of SPCA2/Golgi-dependent Ca^2+^ secretion. As shown in Figure 6, confluent WT MCF7 cells, and Orai1-KO MCF7 cells transfected with plasmids encoding Orai1α-eGFP, Orai1β-eGFP, or empty plasmids were stimulated to induce calcification with an osteogenic medium. Alizarin red staining revealed visible darker extracellular deposits of precipitated Ca^2+^ phosphates in WT MCF7 cells after 7 days of treatment (Fig. 6*A*, yellow arrow), as published elsewhere (18). Consistent with our previous results, quantification of the surface area covered by those crystals revealed that calcification decreased to 12.5% in Orai1-KO MCF7 cells as compared to WT, which was partially recovered to 61.24% by overexpressing Orai1α, but not Orai1β in KO cells (Fig. 6, *A* and *B*; n = 15–22). Results were confirmed with the von Kossa stain, which also highlights Ca^2+^ deposits containing phosphates (Fig. 6*A*). Quantification of the surface area covered by Ca^2+^ phosphate crystals stained in black (Fig. 6*A*, yellow arrow) confirmed that Orai1-KO MCF7 cell cultures present fewer microcalcifications (30.65% as compared to WT), but the microcalcifications were completely recovered by expression of Orai1α, but not Orai1β (Fig. 6, *A* and *C*; n = 12–18). These results indicate that constitutive Ca^2+^ entry mediated by the interaction between SPCA2 and Orai1α, but not Orai1β, participates in the Golgi/secretory pathway leading to the formation of microcalcifications.

## Discussion

SPCA2 plays a crucial role in breast cancer cells and lactation (28). This pump is overexpressed in many breast cancer cells compared to parental tissues, and it has been proposed that oncogenic signaling is triggered by its upregulation (11, 17). Silencing of the Golgi-resident SPCA2 decreased [Ca^2+^]_C_ and abrogated proliferation, migration, tumorigenesis, and calcifications in breast cancer cells (17, 18). Both Orai1 variants are overexpressed in MCF7 and MDA-MB-231 breast cancer cells (11). Their participation in constitutive basal Ca^2+^ entry has been suggested due to its close interaction with SPCA2 (17). It has been proposed that Orai1 is constitutively activated by SPCA2, where the subsequent Ca^2+^ influx is crucial for those SPCA2-dependent processes. Moreover, the overexpressed C-terminal region of SPCA2 promotes NFAT translocation in MCF7 cells, suggesting that SPCA2 also might trigger Orai1-dependent transcription factor activity (17). Two Orai1 variants have been identified in mammalian cells (9); however, direct experimental evidence supporting their participation on constitutive Ca^2+^ entry has not been provided. Thus, we focused our study on the role of Orai1 variants in SPCA2-triggered constitutive Ca^2+^ entry and its role in microcalcification in breast cancer cells. Our results confirm that the absence of Orai1 abrogated constitutive Ca^2+^ entry in MCF7 cells, which is, at least, partially rescued by expression of Orai1α but not when Orai1β is expressed, thus indicating that only the full-length Orai1α variant participates in this mechanism. The lack of Orai1β function might be due to a reduced Orai1β/SPCA2 complex formation (Fig. 1*F*). Previous studies detected the interaction between Orai1 and SPCA2 by immunoprecipitation and pull-down assays, indicating that both proteins form a complex (17, 19). Our immunoprecipitation experiments confirmed previous results and showed a reduced interaction between Orai1β and SPCA2 (Fig. 1*F*). However, these methods cannot discriminate between direct or indirect protein interactions. FRET analysis (Fig. 3) strongly supports direct interaction between Orai1 variants and SPCA2. Moreover, FRET experiments confirmed the residual weak SPCA2/Orai1β interaction. Orai1-KO cells expressing Orai1β showed reduced FRET in both the PM and the cytosol, indicating an increased distance between SPCA2 and Orai1β, and suggesting a reduced interaction (Fig. 3*B*). Feng *et al.* proposed a sequential interaction model where the N-terminus first, and later the C-terminus of Orai1 binds to the N-terminus of SPCA2 (17). The C-terminus of SPCA2 is then exposed as a result of the recruitment of both ends, interacting back with an unknown region of Orai1, which, in turn, switches, to the active state. Regarding our results, we propose that the lack of the N-terminal 63 or 70 amino acids in Orai1β impair the activation sequence in the initial step, reducing the transition to the next step, where the C-terminus of SPCA2 is exposed. Although speculative, this might explain the reduced Orai1β/SPCA2 interaction as compared to that for Orai1α. Thus, both channels form reduced complexes, but with inactive Orai1β channels. Surprisingly, FRET analysis revealed that only a small fraction of both SPCA2 and Orai1α binds with each other, suggesting that the number of participating proteins might be limited by a regulatory mechanism that promotes SPCA2/Orai1α complex formation, thus preventing Ca^2+^ overload and cytotoxicity (29, 30). Cross. *et al.* proposed that SPCA2 regulates cell surface trafficking of Orai1 in mammary epithelial SCp2 cells. Silencing one of both proteins altered the expression pattern of the other protein, demonstrating their interdependence for their correct subcellular location (19). Our colocalization experiments showed no differences in the distribution of both Orai1 variants (Fig. 2). Thus, we propose that the conserved SPCA2/Orai1β interaction might be necessary for maintaining their proper location. Feng. *et al.* mapped the interacting region with SPCA2 as the region 48 to 91 at the N-terminal region of Orai1α (17). The reduced interaction with SPCA2 of the short Orai1 form, Orai1β, might restrict further the binding site to amino acids 48 to 63 or 48 to 70. Unexpectedly, our experiments using the O1Δ1→38 mutant showed abrogated constitutive Ca^2+^ entry and reduced SPCA2 complex formation to a similar extent as Orai1β, thus reporting that the N-terminal fragment between amino acids 1 and 38 is essential for the interaction with SPCA2. This contradicts some Feng *et al.* results, since they could not pull-down SPCA2 using a flag-tagged peptide containing the first 47 aa of Orai1α (17). Such discrepancy is difficult to explain, but the fact that we used the complete Orai1β to immunoprecipitate SPCA2 instead of a peptide must be a possible reason. Moreover, our results indicate that Orai1αΔ1-38 fails to activate constitutive Ca^2+^ entry, indicating the importance of this region in this mechanism.

The Orai1α N terminal 38 amino acids comprise a variety of functional regions and sites, including the phosphorylatable serine residues 27 and 30. However, our results indicate that the PKC phosphorylation sites, Ser-27 and Ser-30, are not involved in the interaction with SPCA2, since the Orai1α O1AA and O1DD mutants did not exhibit altered constitutive Ca^2+^ entry. We also discarded the participation of RyR in the control of basal [Ca^2+^]_C_, since high concentrations of ryanodine, which result in inhibition of Ca^2+^ release (31), did not alter the basal [Ca^2+^]_C_ or constitutive Ca^2+^ entry (Fig. 5, *D* and *E*).

Finally, we have studied the functional relevance of the Orai1 variants on breast cancer microcalcifications through the interaction with SPCA2. Intense research has been done to describe the mechanisms underlying the increased Ca^2+^ depositions found in breast cancer patients (27). Ca^2+^ oxalate frequently appears in benign tumors, whereas Ca^2+^ phosphates appear in both benign and malignant tumors as hydroxyapatite (27). We used alizarin red at a pH of 4.2 and von Kossa staining, which stain Ca^2+^ phosphates but not oxalates (32). As compared to WT MCF7 cells, we found a decreased calcification in Orai1-KO MCF7 cells that was rescued by expression of the long Orai1 variant, Orai1α, but could not be rescued by overexpressing Orai1β, indicating that only Orai1α plays a functional role in MCF7-induced microcalcification.

Summarizing, our results indicate that only Orai1α plays a functional role in the conduction of constitutive Ca^2+^ entry through the interaction with SPCA2 in MCF7 cells, while Orai1β might be a modulator of this Ca^2+^ entry mechanism in these cells. The N-terminal region between amino acids 1 and 38 plays an essential role in the interaction with SPCA2 and deletion of this region impaired the Orai1α/SPCA2 interaction to a similar extent as the interaction observed between SPCA2 and the short Orai1 form, Orai1β, lacking 63 or 70 N-terminal amino acids. Constitutive Ca^2+^ entry is believed to be important for Ca^2+^ incorporation into the milk by mammary epithelial cells (28). The participation of Orai1α in constitutive Ca^2+^ entry has been reported on *in vitro* cellular models of lactation (19, 33), and mammary alveolar epithelial cells from lactating Orai1 KO mice are less efficient in the transfer of Ca^2+^ ions into the milk (34). Through the activation of constitutive Ca^2+^ entry in MCF7 breast cancer cells, Orai1α incorporates extracellular Ca^2+^ into the cytosol, which is subsequently transferred to the Golgi by SPCA2, to be finally secreted to the extracellular compartment as insoluble Ca^2+^ phosphates, resulting in calcification. Therefore, our results provide evidence for the role of Orai1α in breast cancer-induced calcification.

## Experimental procedures

### Reagents

Fura-2 acetoxymethyl ester (Fura2-AM) and cell culture reagents such as Dulbecco's modified Eagle's medium (DMEM), fetal bovine serum, penicillin/streptomycin, Pierce bicinchoninic acid protein assay kit and SuperSignal West Dura detection reagent and trypsin were purchased from Thermo Fisher Scientific. Ryanodine, ammonium persulfate, EDTA, IGEPAL CA-630, protein A agarose beads, sodium azide, SDS, *N*, *N*, *N*', *N*' - tetramethylethylenediamine, Tween 20, bovine serum albumin fraction V (BSA), Hepes, anti-Orai1 (cat. O8264, epitope: amino acids 288–301 of human Orai1), anti-β-actin antibody (cat. A2066), alizarin red, aluminum sulfate hydrate, ammonium hydroxide, dexamethasone, L-ascorbic acid, sodium phosphate (Na_2_HPO_4_ and NaH_2_PO_4_), silver nitrate, sodium thiosulfate and EGTA were purchased from Sigma-Aldrich. DharmaFECT cell transfection reagent was purchased from Cultek. Bis-acrylamide was purchased from Thermo Fisher Scientific. Complete EDTA-free protease inhibitor cocktail was purchased from Roche Diagnostics GmbH. Nitrocellulose membrane and Whatman paper were purchased from Amersham Biosciences. Anti-mCherry antibody (cat. ab125096) was purchased from Abcam. Horseradish peroxidase–conjugated goat anti-mouse and goat anti-rabbit secondary antibodies were purchased from Jackson Laboratories. Goat anti-rabbit StarBright Blue 700 fluorescence secondary antibody was purchased from Bio-Rad. Reagents for calcification experiments such as fast nuclear red and Human BMP-2 recombinant protein (cat. 10432565) were purchased from Thermo Fisher Scientific. MCF7 WT and CRISP-generated Orai1 KO cell lines were a gift from Rajesh Bhardwaj (Hediger Membrane Transport Discovery Lab, University of Bern, Switzerland). pLKO.3G backbone expressing free eGFP was a gift from Christophe Benoist & Diane Mathis (Addgene plasmid # 14748; http://n2t.net/addgene:14,748; RRID:Addgene_14748). Orai1α-eGFP and Orai1β-eGFP plasmids were a gift from Mohamed Trebak (Department of Pharmacology and Chemical Biology, University of Pittsburgh, USA). pEYFP-Orai1α N-terminal deletion mutant Orai1α ΔN1→38 (O1Δ1→38) was a gift from Christoph Romanin (Institute of Biophysics, Johannes Kepler University Linz, Austria). Double mutant lacking two phosphorylation sites Flag-Orai1-S27A/S30A (Orai1-AA) and double mutant phosphomimetic Flag-Orai1-S27D/S30D (Orai1-DD) were a gift from Agustin Guerrero (CINVESTAV, Mexico), Single mutant Orai1-S34A-GFP (Orai1-34) was a gift from Javier Martin-Romero (University of Extremadura), mCherry-SPCA2 plasmid was a gift from Peter Vangheluwe (Department of Cellular and Molecular Medicine, KU Leuven, Belgium).

### Cell culture

Cells were cultured in DMEM supplemented with 10% (v/v) fetal bovine serum and 100 U/ml penicillin/streptomycin under 5% CO_2_ and 95% humid atmosphere at 37 °C. Transient plasmid transfection was performed with DharmaFECT kb reagent in 80% confluent cells in the presence of the abovementioned plasmids following the manufacturer's instructions. For calcification experiments, transfection was performed in confluent cell monolayers. Experiments were carried out 24 h after transfection.

### Assessment of cytosolic free-Ca^2+^ concentration ([Ca^2+^]_c_)

Cultured cells (5 × 10^5^ cells) were seeded on coverslips, transfected with Orai1-GFP or empty pLKO.3G plasmids and loaded with fura-2 after 24 h by incubation with 5 μM fura-2/AM for 30 min at 37 °C. Cells were mounted on a perfusion chamber and placed on the stage of an epifluorescence inverted microscope (Nikon Eclipse Ti2) with an image acquisition and analysis system for video microscopy (NIS-Elements Imaging Software v.5.02.00, https://www.microscope.healthcare.nikon.com/es_EU/products/software/nis-elements, Nikon, Amsterdam, The Netherlands). Cells were continuously superfused at room temperature with Hepes-buffered saline containing (in mM) 125 NaCl, 5 KCl, 1 MgCl_2_, 5 glucose, and 25 Hepes, pH 7.4, supplemented with 0.1% (w/v) BSA. Cells were examined at 40× magnification (Nikon CFI S FLUOR 40 × Oil) and were alternatively excited with light from a xenon lamp passed through a high-speed monochromator Optoscan ELE 450 (Cairn Research) at 340/380 nm. Fluorescence emission at 510 nm was detected using a cooled digital sCMOS camera PCO Panda 4.2 (Excelitas PCO GmbH) and recorded using NIS-Elements AR software (Nikon, Amsterdam, The Netherlands). eGFP positive transfected cells were selected with a ROI, fluorescence ratios (F340/F380) were calculated pixel by pixel, and the data were presented as ΔF340/F380 as described previously (13). Constitutive Ca^2+^ entry was estimated as the area under the curve measured as the integral of the rise in fura-2 fluorescence ratio after the addition of extracellular Ca^2+^ ([Ca^2+^]_O_) and taking a sample every second. The increase and decrease constants were estimated using the following equation:

$$b=\frac{\sum \left(x-\bar{x}\right)\left(y-ȳ\right)}{\sum {\left(x-\bar{x}\right)}^{2}}$$

Were b is the increase constant (for positive slopes) or the decrease constant (for negative slopes), x is the time (in seconds) and y is the Δfura-2340/380 nm fluorescence ratio. $\bar{x}$ and ȳ represent averaged x and y values, respectively.

### Immunoprecipitation and Western blotting

For immunoprecipitation experiments, 4 × 10^6^ MCF7 KO cells were transfected with Orai1-GFP and mCherry-SPCA2 plasmids. Cells were lysed 24 h after with IGEPAL CA-630 buffer (137 mM NaCl, 20 mM Tris-Base, 2 mM EDTA, 10% glycerol, 1% IGEPAL CA-630, pH 8) supplemented with 1 mM Na_3_VO_4_ and complete EDTA-free protease inhibitor tablets. The insoluble cellular fraction was removed by centrifugation and the protein concentration was assessed by bicinchoninic acid assay and adjusted to 1 mg/ml total protein concentration for all samples. Cell lysates were incubated with 2 μg of anti-Orai1 antibody and 20 μl of protein A-agarose under continuous rotation at 4 °C overnight. Cell lysates and immunoprecipitates were resolved in 10% SDS-PAGE gels and transferred onto nitrocellulose membranes for subsequent antibody probing. Membranes were blocked with 10% BSA in Tris-buffered saline (TBS) supplemented with 0.1% Tween-20 (TBST) for 1 h at room temperature. Membranes were incubated for 1 h with anti-Orai1 (1:1000), anti-mCherry (1:1000), or anti-β-actin (1:2000) antibody reconstituted in 3% BSA/TBST to detect proteins of interest. Membranes were washed with TBST and incubated for 1 h with horseradish peroxidase–conjugated goat anti-mouse or goat anti-rabbit antibody diluted 1:10,000 in TBST in the case of chemiluminescence or with goat anti-rabbit StarBright Blue 700 fluorescence secondary antibody (1:2500). Washed membranes were then exposed to enhanced chemiluminescence reagents for 5 min. Chemiluminescence was documented with a ChemiDoc Imaging System (Bio-Rad), and the density of bands was quantified with ImageJ software (NIH). Immunoprecipitated mCherry-SPCA2 data were normalized against the total mCherry-SPCA2 (input) that was subsequently normalized against the immunoprecipitated Orai1. For whole cell lysate Western blots, β-actin was used as loading control to verify an equal amount of total protein loaded in each lane.

### Confocal microscopy

MCF-7 KO (5 × 10^5^ cells) were seeded in round 25 mm coverslips and cultured in six-well plates. Confluent cells (60%) were transfected with Orai1α-eGFP, Orai1β-eGFP, and mCherry-SPCA2 tagged plasmids. Cells were imaged 24 h post transfection using a confocal microscope (LSM900; Zeiss) with 63× oil immersion objective, using an image acquisition and analysis system for video microscopy (ZEN Software; https://www.zeiss.com/microscopy/es/productos/software/zeiss-zen.html; Zeiss). Fluorescence was detected by an Airyscan detector. The pinhole was set at 1 Airy Unit (23.5099 pixels per micron resolution) and no line averaging was used. Data were recorded with Zen Blue 3.4 acquisition and analysis software from Zeiss (https://www.zeiss.com/microscopy/es/productos/software/zeiss-zen.html). Images were analyzed with ImageJ software (https://imagej.net/ij/, NIH). Colocalization was performed on selected ROIs around the whole cell or the PM and expressed as mean Pearson's correlation coefficient ± S.E.M.

### Acceptor photobleaching FRET

Acceptor photobleaching FRET experiments on MCF-7 KO cells transfected with free eGFP Orai1α-eGFP or Orai1β-eGFP and mCherry-SPCA2 plasmids were carried out with the abovementioned confocal microscopy setup. Free eGFP was used as a negative control since it should not produce FRET when combined with mCherry SPCA2. Cells seeded in round 25 mm coverslips were mounted in a chamber and superfused with Hepes-buffered saline. Single-cell FRET efficiencies were obtained registering the progressive increase in the GFP (donor, 488/509 nm excitation/emission) fluorescence after mCherry (acceptor, 587/610 nm) photobleaching along 13 cycles. Within each ROI, the fluorescence intensities of the donor and acceptor were measured independently. Each cycle contained three steps, a previous GFP excitation/fluorescence registration at 488/509 nm respectively, followed by a mCherry excitation/fluorescence registration at 587/610 nm. As a final step, mCherry was bleached by increasing the 587 nm laser power to ten times its original value, thereby gradually reducing their fluorescence intensity by 30 to 40% each time. FRET efficiencies were expressed as the maximal increase of the GFP donor fluorescence in percentage after complete mCherry bleaching. To measure FRET, nine ROIs were placed on each cell, three in the cytosolic, Golgi, and PM regions. Usually, only one, or a small number of the nine ROIs showed signs of FRET in each measurement. As a result, the frequency of FRET events in each subcellular region was determined as follows: each of the nine ROIs received a value of 1 or 0 depending on whether FRET was present or absent. The total number of 1 values was determined for all measured Orai1α or Orai1β transfected cells per day. Subsequently, the number of 1 values seen in each subcellular region against the total number of FRET events recorded in all three regions was calculated as percentage to determine the frequency of FRET events. FRET frequencies obtained for each day of experiment were averaged and represented as mean FRET frequency (%) for each subcellular region ± SEM.

### Induction of extracellular calcifications

MCF7 WT and KO (6 × 10^5^ cells) were seeded in 24-well plate in regular full DMEM media and transfected next day at 90% confluency. To induce calcification, 100% confluent cells were cultured in full DMEM supplemented with 200 ng/ml BMP-2 recombinant protein, 10 nM dexamethasone, 50 μg/ml ascorbic acid, and 5 mM of inorganic phosphate source (4 parts of Na_2_HPO_4_/1 part of NaH_2_PO_4_, pH 7.2) for 7 days. Calcification media were replaced every 2 to 3 days.

### Specific staining of calcium precipitates

Cells were washed in PBS and fixed in 4% paraformaldehyde for 30 min. Cultured cells were stained with alizarin Red or Von Kossa dyes to highlight tissue calcium deposits. For alizarin red staining, fixed cells were stained with 2% alizarin red solution adjusted with ammonium hydroxide to pH 4.4 for 5 min and washed in PBS. For Von Kossa staining, fixed cells were incubated in 1% silver nitrate under a UV lamp (300 nm) for 5 min, washed with bi-distilled water, and incubated with 5% sodium thiosulfate for 5 min to remove unreacted silver. Cells were counterstained with 0.1% nuclear fast red/5% aluminum sulfate solution for 5 min and finally washed with bi-distilled water.

### Statistical analysis

All data are presented as the mean ± standard error of mean (SEM). Analysis of statistical significance was performed using GraphPad Prism v.8.4.3 (https://www.graphpad.com/, GraphPad Software, San Diego, CA, USA). Kruskal–Wallis test combined with Dunn's *post hoc* test were used to compare the different experimental groups. For comparison between two groups, the unpaired-*t* test and the Mann–Whitney U test was used. All data with *p* < 0.05 was deemed significant.

## Data availability

The experimental data reported in this work are available from the corresponding authors upon reasonable request.

## Supporting information

This article contains supporting information.

## Conflict of interest

The authors declare that they have no conflicts of interest with the contents of this article.
