# Supplementary material for: Differential functional role of Orai1 variants in constitutive Ca2+ entry and calcification in luminal breast cancer cells
Source: J Biol Chem. 2024 Sep 18;300(10):107786. doi: 10.1016/j.jbc.2024.107786 (PMC11736007; doi:10.1016/j.jbc.2024.107786)
Supplement: Supplemental material [file mmc1.docx]

*SUPPLEMENTAL MATERIAL*

**Differential functional role of Orai1 variants in constitutive Ca^2+^ entry and calcification in luminal breast cancer cells**

Alejandro Berna-Erro, Jose Javier Lopez, Isaac Jardin, Jose Sanchez-Collado, Gines M. Salido, Juan A. Rosado.

Department of Physiology (Cellular Physiology Research Group), Institute of Molecular Pathology Biomarkers (IMPB), University of Extremadura, 10003-Caceres, Spain.

CONTENT:

Figure S1: Alignment of human Orai1α, Orai1β and the Orai1α mutants Orai1αS27/30A, Orai1αS27/30D and Orai1αΔ1-38.

Figure S2 Role of Orai1α phosphorylation at Ser-34 in constitutive Ca^2+^ entry in MCF7 cells.

Figure S3: Uncropped gel images Figure 1B, F; Figure 4B, F; Figure 5B; Figure S2.

**Figure S1**


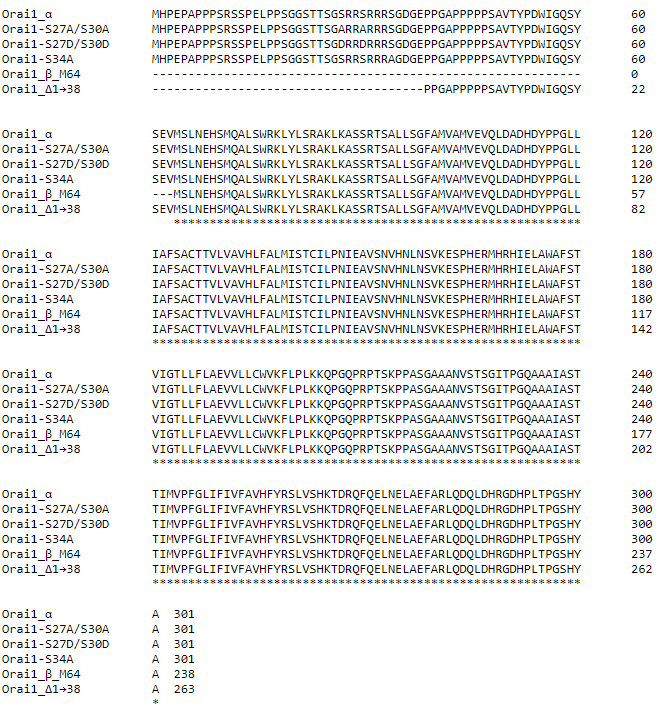


Figure S1: **Alignment of human Orai1α, Orai1β and the Orai1α mutants Orai1αS27/30A, Orai1αS27/30D and Orai1αΔ1-38.**

**Figure S2**


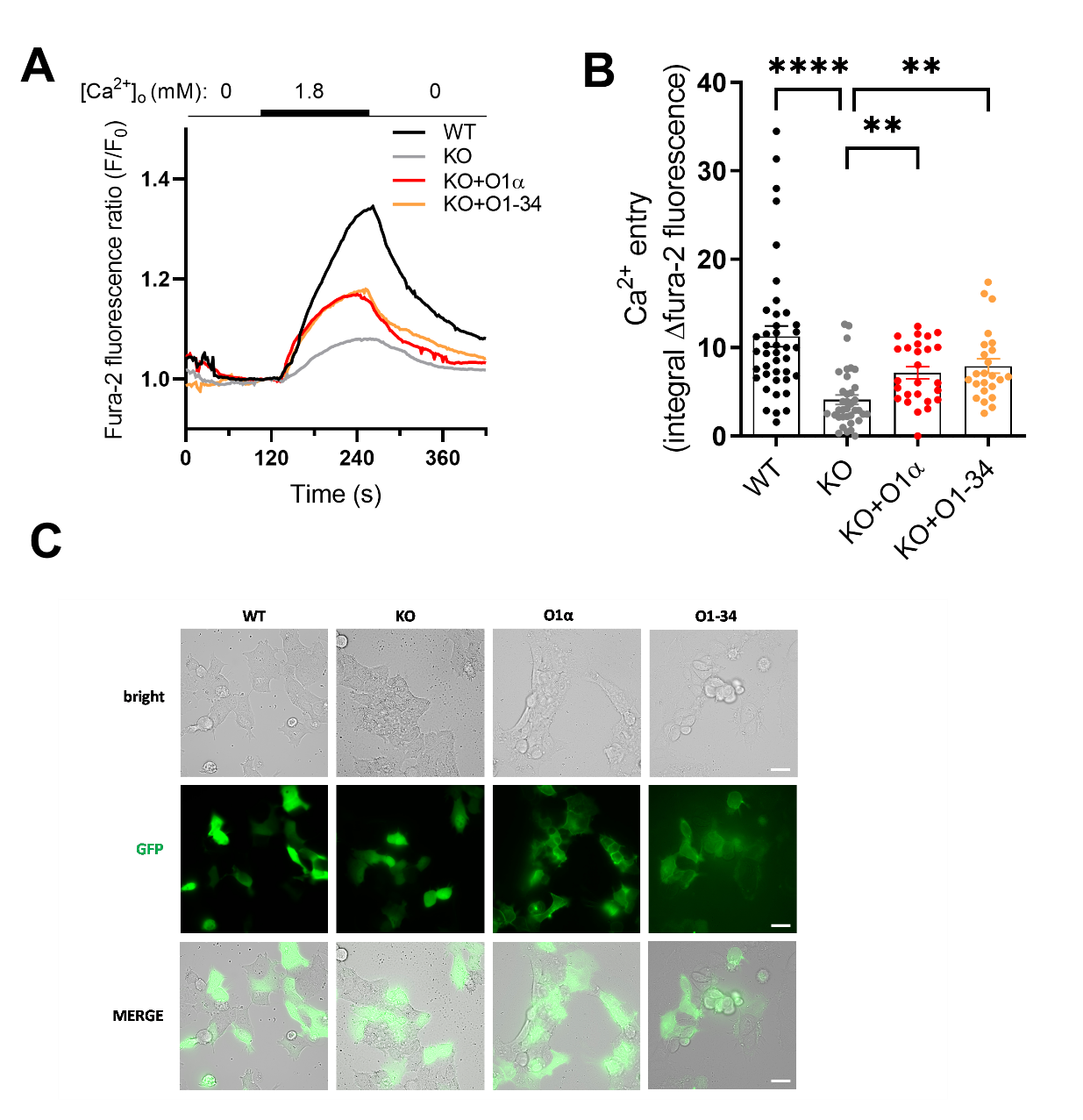


Figure S2 **Role of Orai1α phosphorylation at Ser-34 in constitutive Ca^2+^ entry in MCF7 cells.** *A-C*, Wild type MCF7 cells (WT) and Orai1-KO MCF7 cells (KO) were transfected with pLKO.3G backbone expressing free eGFP, while KO cells transfected with CMV promoter plasmids either for Orai1α (O1α) or Orai1α-S34A mutant (O1-34), and subsequently loaded with fura-2. *A*, Fura-2-loaded cells were perfused with a Ca^2+^-free HBS (100 µM EGTA added), followed by perfusion with HBS containing 1.8 mM Ca^2+^ and then further perfused with a Ca^2+^-free HBS (1 mM EGTA added). Graphs represent mean values. *B*, Quantification of Ca^2+^ entry was determined as described in materials and methods. Bar graphs are represented as mean ± SEM and expressed as the integral of the rise in fura-2 fluorescence ratio after the addition of extracellular Ca^2+^ and taking a sample every second. From left to right, n = 41, 36, 26, and 24; n values correspond to individual cells. Data were statistically analyzed using Kruskal–Wallis test with multiple comparisons (Dunn´s test). **P < 0.01 and ****P < 0.0001. *C*, Representative images of MCF7 transfected cells observed at bright field (bright), GFP fluorescence channel (GFP) or merged (MERGE) used for imaging experiments. Scale bar = 30 μm.

Figure S3 **Uncropped blots.**

Figure 1B


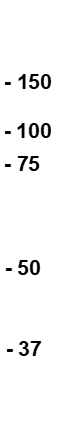


**kDa**

**kDa**


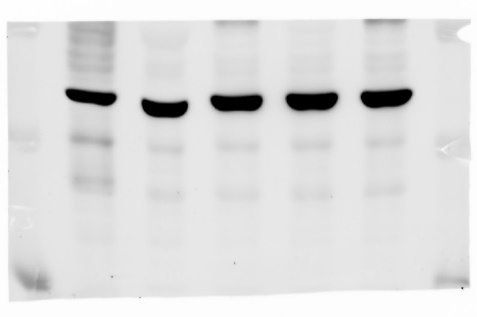


WB: anti-β-actin


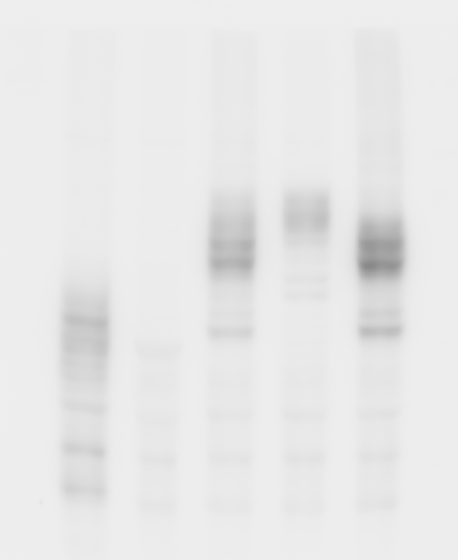


WB: anti-Orai1

actin


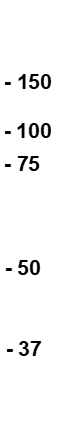


**- 25**

Figure 1F


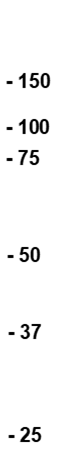


**kDa**

WB: anti-Orai1 IP

actin

**kDa**

WB: anti-mCherry IP

actin

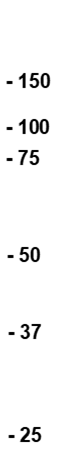


WB: anti-mCherry and anti-β-actin Lysates

actin

**kDa**


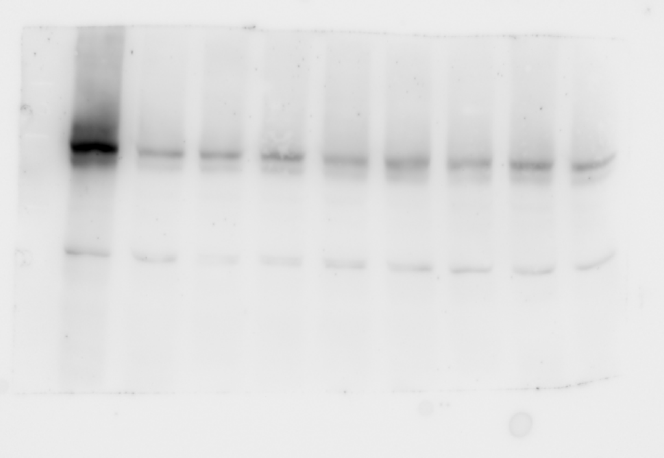


**- 25**

**- 37**

**- 50**

**- 75**

**- 100**

**- 250**

**- 150**

Figure 4B

**kDa**

WB: anti-Orai1

actin

**- 150**


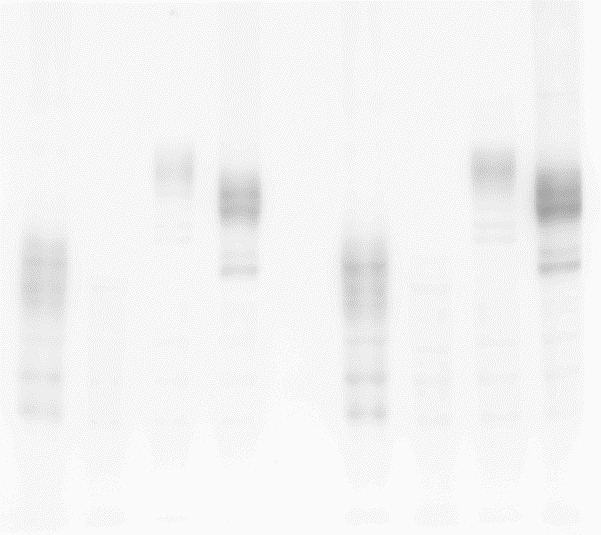


WB: anti-β-actin

**- 50**

**- 75**

**- 100**


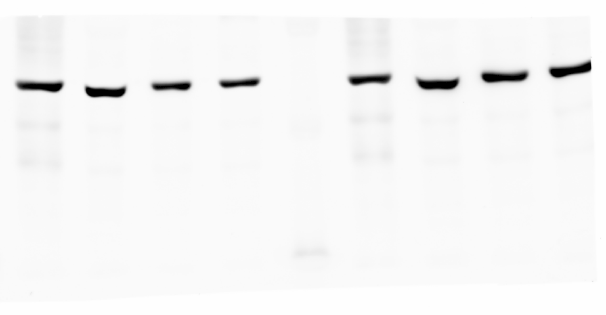


**- 50**

**- 37**

**- 25**

**- 37**

**- 25**

Figure 4F


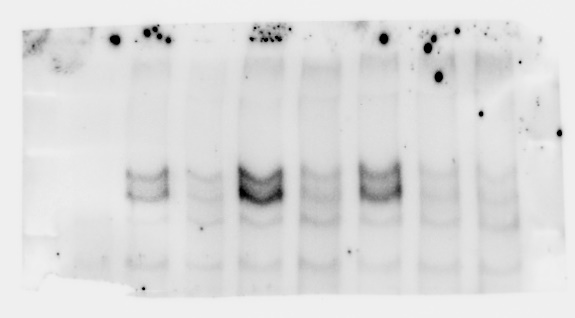


WB: anti-mCherry

actin

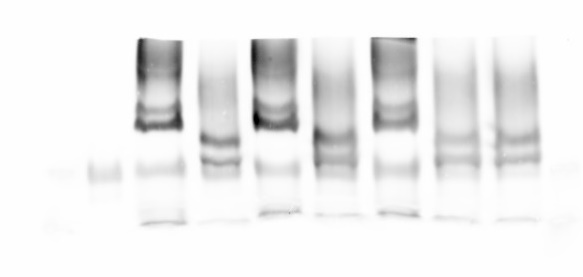


WB: anti-Orai1

**- 75**

**- 50**

**- 250**

**- 150**

**- 37**

**- 100**

**- 75**

WB: anti-mCherry Lysates

actin

WB: anti-β-actin Lysates

actin

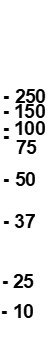


**- 250**

**75**

**-**

**- 100**

**- 150**

**- 50**

**- 37**

**- 25**

**- 10**

Figure 5B


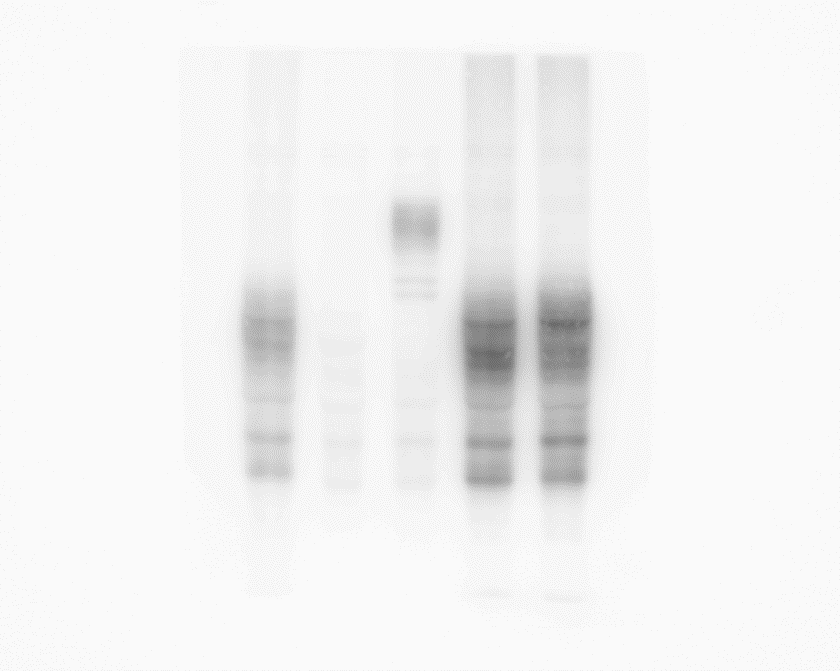


WB: anti-Orai1

actin


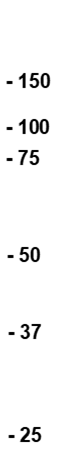

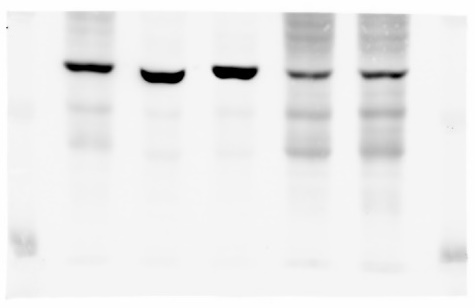

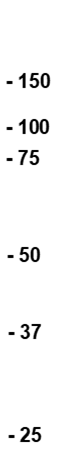


**- 25**

**- 25**

**kDa**

WB: anti-β-actin

**kDa**
